# Phenomenology and Physiology of Tacrolimus Induced Tremor

**DOI:** 10.5334/tohm.725

**Published:** 2023-01-30

**Authors:** Aparna Wagle Shukla, Caroline Lunny, Ibrahim Hisham, Jackson Cagle, Joyce Malea, Alfonso Santos, Ashutosh M. Shukla

**Affiliations:** 1 Department of Neurology, Fixel Institute for Neurological Diseases, University of Florida, Gainesville, Florida, United States of America; 2 Division of Nephrology, Department of Medicine, University of Florida, Gainesville, Florida, United States of America; 3 North Florida South Georgia Veteran Healthcare System, Gainesville, Florida, United States of America

**Keywords:** tacrolimus, tremor, tacrolimus induced tremor, physiology, limb cooling, cooling, drug induced tremor, adaptive device

## Abstract

**Background:**

Tacrolimus is a potent immunosuppressant drug commonly used after solid organ transplant surgery. The use of this drug is frequently associated with the emergence of tremors. There is little information on the clinical and physiological characteristics of tacrolimus-induced tremors. Characterizing these tremors is essential as they can promote the development of specific therapies.

**Methods:**

We describe four patients placed on tacrolimus immunosuppressant therapy following kidney transplant surgery and who developed tremors impacting their daily functional activities. We describe the clinical and physiological characteristics of the tremor and the response generated after a limb cooling test.

**Results:**

A postural and kinetic tremor mainly involving the distal hands was observed in our cohort. In the accelerometer-based assessment, the tremor amplitude was noted to be mild to moderate, and the frequency was 5–6 Hz. Cooling the forearm and the hand led to a temporary albeit significant reduction of tremor amplitude (p = 0.03). Limb cooling lowered the tremor frequency by 1 Hz in two patients with no change in the other two patients, and the statistical comparison was not significant (p > 0.05).

**Conclusions:**

Limb cooling may be pursued as a therapeutic option for addressing tacrolimus-induced tremor, as the patients in our cohort benefitted from temporary tremor suppression.

## Introduction

Tremor is observed to emerge with a variety of commonly used prescription drugs, including bronchodilators, antiarrhythmics, antidepressants, mood stabilizers, neuroleptics, chemotherapeutics, and immunosuppressants [1,2]. Among the immunosuppressants category, tacrolimus is particularly important as it has been observed to frequently lead to tremors.

Tacrolimus (or FK506), a calcineurin inhibitor, is the cornerstone immunosuppressive agent after a solid organ transplant [3,4]. Over the last few decades, solid organ transplant surgeries have shown a trend for a steady incline in number. For example, in 2018, according to the US Renal Data System report, over 22,000 kidney transplant surgeries were performed [5]. With the availability of tacrolimus, the graft rejection rates have significantly declined and the clinical outcomes have improved tremendously [6,7,8,9]. However, several neurological complications, including tremors, seizures, delirium, ataxia, and posterior reversible encephalopathy syndrome, remain as important causes of concern [3,9].

Tacrolimus intake has reportedly been associated with tremor affecting more than 50–70% of patients [10,11]. Despite such high numbers, the phenomenological and physiological characteristics of tacrolimus-induced tremors are unknown. Such knowledge is imperative for developing specific treatments. We ascertained the clinical nature of tacrolimus-induced tremors, the body distribution pattern, and the response to different motor activation tasks such as postural elevation of arms and simple kinetic tasks such as writing and drawing in a series of kidney transplant patients referred to our clinic. We determined the physiological characteristics such as frequency, rhythmicity, amplitude, and response to limb cooling.

## Methods

We conducted a prospective observatory study after obtaining the regulatory approvals from the University of Florida Institutional Review Board. Consecutive kidney transplant recepients with tremor were referred for neurology evaluations. The clinical diagnosis of tremor was confirmed by a movement disorder neurologist folowing the MDS criteria. The diagnosis of the tremor was confirmed by a movement disorder neurologist following the Movement Disorders Society criteria [12]. We employed an IRB-approved protocol to study the patients. Clinical assessments were performed primarily using items from the Fahn Tolosa Marin tremor rating scale [13]. (Table 1). We also used the dot approximation task, an item included in The Essential Tremor Rating Assessment Scale [14]. Participants were seated comfortably in an upright chair with a backrest and headrest. Tremor was observed clinically for assessment of rest, postural and kinetic components. For the rest component, arms and hands were resting on the armrest with the wrist allowed to dangle unsupported over the edge of the supportive surface for 60 seconds. The postural tremor was observed with arms and hands outstretched at 90 degrees from vertical, keeping parallel to the ground with the palms facing down and the fingers spread slightly apart from each other. The kinetic tremor was assessed with standard instructions for spiral drawing, line drawing, dot approximation, and writing tasks.

**Table 1:** Clinical characteristics of patients with tacrolimus induced tremor.


	PATIENT 1	PATIENT 2	PATIENT 3	PATIENT 4

**Age in years**	63	57	73	72

**Sex**	male	female	male	male

**Diagnosis**	ESRD secondary to type II diabetes	ESRD secondary to ADPKD	ESRD secondary to FSGS	ESRD secondary to glomerulonephritis

**Disease duration**	6 years	8 months	15 years	10 years

**Tacrolimus Dose in mg/day**	8	4	2	3.5

**Tacrolimus Level (nG/mL)**	5.4	11	5.1	3.3

**Onset time for tremor after tacrolimus**	one week	few weeks	three weeks	two weeks

**Tremor Duration**	4 months	3 months	11 years	10 years

**Functional activity most important**	writing	jewelry crafting	writing	eating

**Rating of task**				

**speaking**	0	0	0	0

**feeding**	2	2	2	2

**bringing liquids to mouth**	2	3	2	2

**hygiene**	1	3	2	1

**dressing**	1	2	2	1

**writing**	2	2	2	2

**working**	2	4	2	1

**Activities of daily living total score**	10	16	12	9

**Physical examination**				

**rest tremor score**	0	0	1	0

**posture tremor score**	1	1	3	1

**action/kinetic tremor score**	2	1	2	2

**dot approximation task score**	1	1.5	2	2

**spiral drawing task score (worst)**	1	0	2	1

**line drawing score**	0	0	1	1

**handwriting score**	1	0	2	1

**TRS Total Score**	20	24	34	21


ESRD: End stage renal disease. ADPKD: Autosomal Dominant Polycystic Kidney Disease. FSGS: Focal segmental glomerulosclerosis.

**Figure 1:**
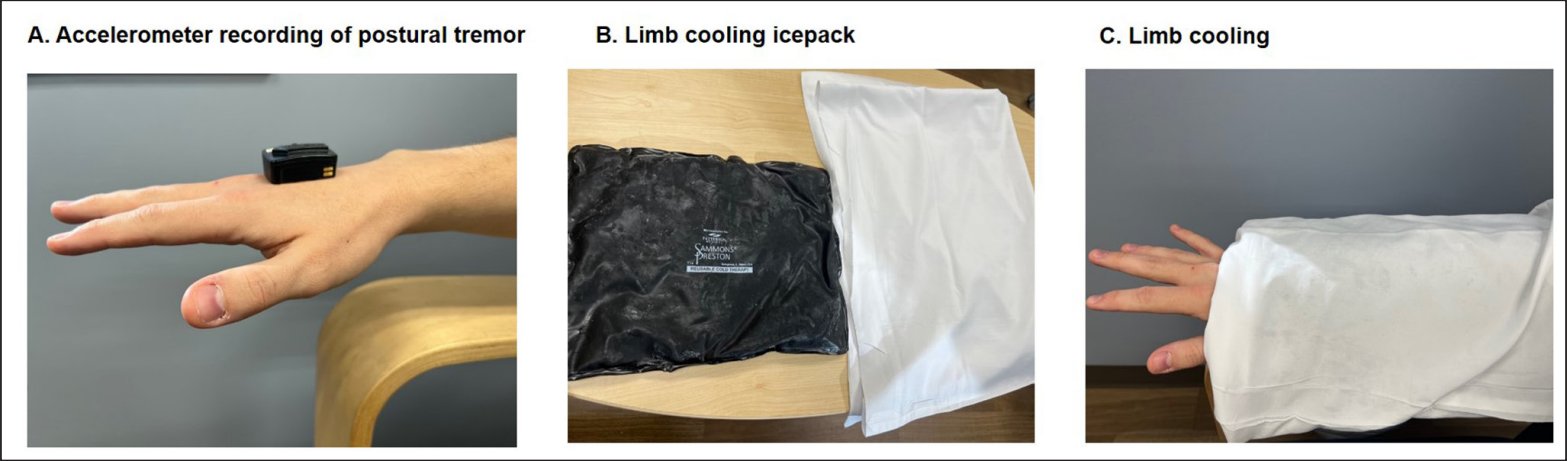
**A.** Placement of accelerometer (Trigno sensor) on the dorsum of the hand, sensor placed 1 cm distance proximal to the third metacarpophalangeal joint. The patient maintained a steady extended posture. **B.** Reusable Icepack from Patterson Medical was used. **C.** There were two icepacks placed in two pillowcases that were wrapped around the forearm and the hand. Tremor was recorded immediately after cooling while the patient maintained a steady extended posture and the accelerometer placed on the dorsum of the hand.

**Figure 2:**
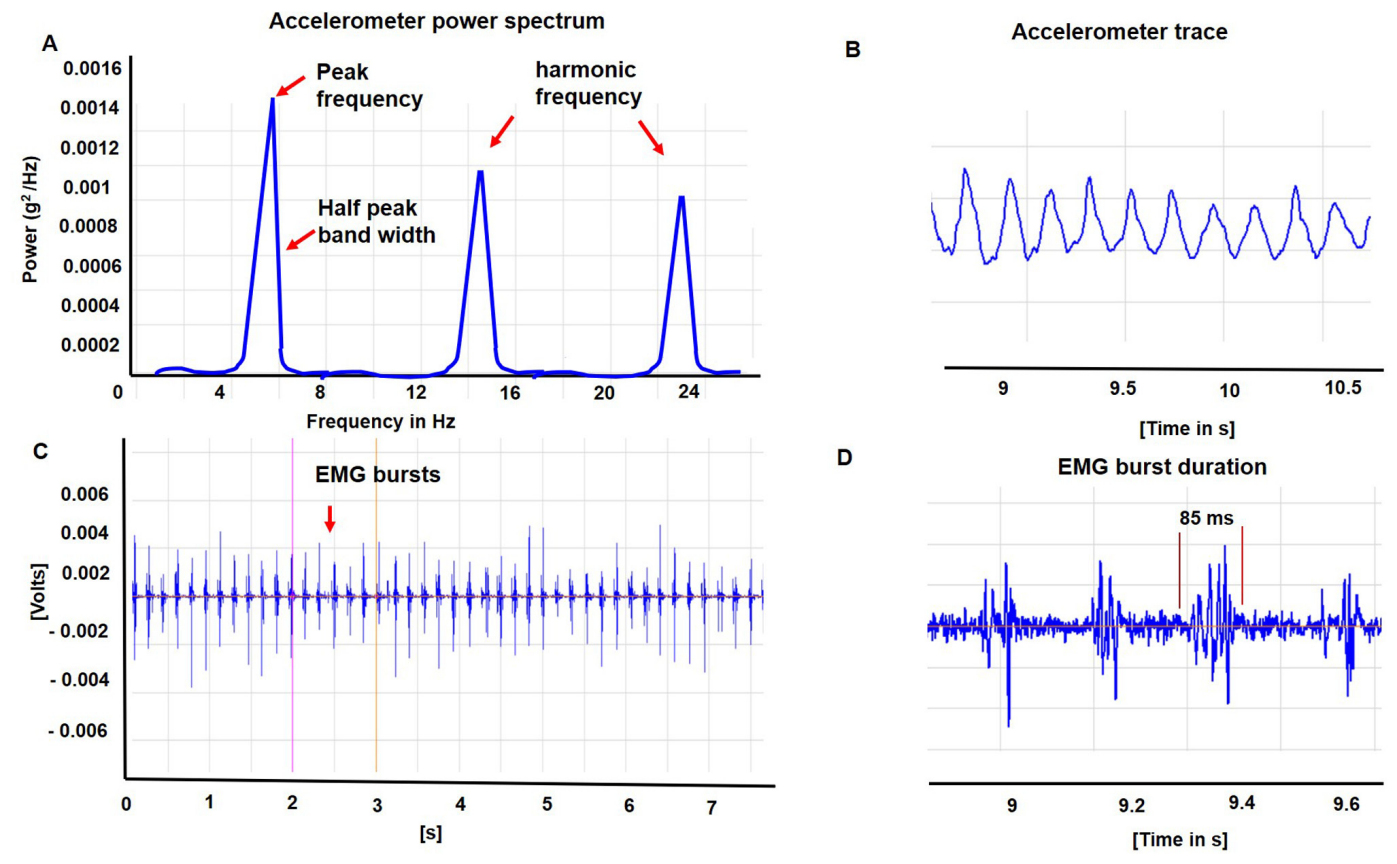
**A.** Power spectrum analysis of accelerometer recording from patient 3. Peak frequency and harmonic frequencies are depicted. The unit for amplitude is g^2^/Hz. The frequency unit is hertz. Half-peak bandwidth shown in the figure was calculated as the width of the spectral peak at one-half the peak amplitude in the power spectrum. High-frequency harmonic frequencies are shown **B.** Accelerometer tracing reveals that the oscillations were rhythmic but the waveforms were not necessarily sinusoidal. **C.** EMG bursts recorded from extensor carpi radialis muscle of patient 3. **D.** EMG recording of extensor carpi radialis muscle revealing 75–85 ms as the average duration of bursts.

**Figure 3:**
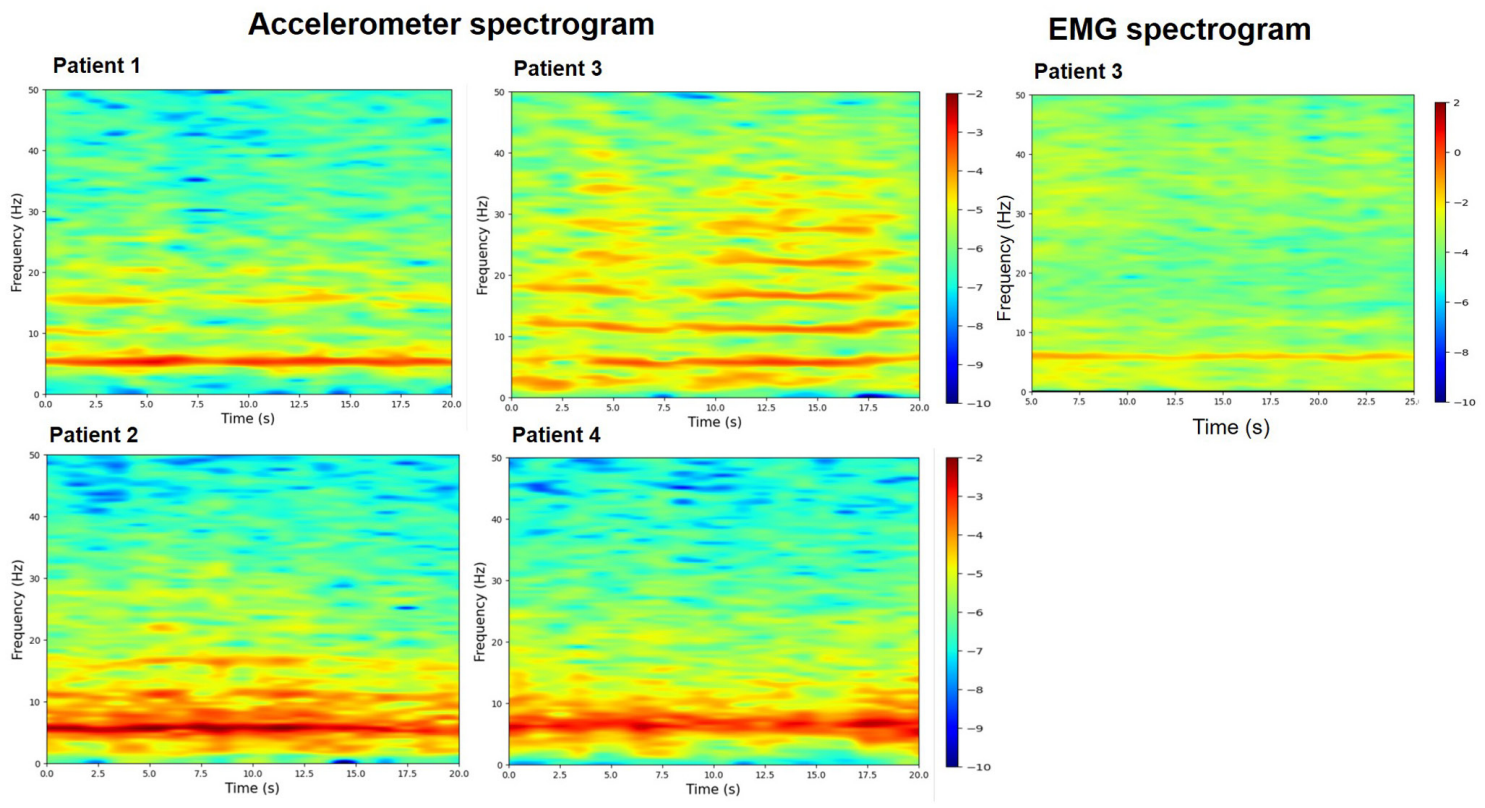
Plot revealing time-frequency spectrogram of tremor power for individual patients recorded with accelerometer and EMG. The fundamental tremor frequency remained stable across time.

We recorded the physiology when maintaining a steady posture (Figure 1A). We used the Trigno™ Wireless system (Delsys, Inc., Massachusetts) consisting of triaxial orthogonal accelerometers to record the postural component of tremor. Sensors were mounted on the dorsum of the most affected hand at a 1 cm distance, proximal to the third metacarpophalangeal joint, to capture the accelerometer data. We ensured that there was consistent sensor placement across individuals. Sensors were also mounted over the flexor carpi ulnaris, flexor carpi radialis, extensor carpi ulnaris, and extensor carpi radialis muscles of the most affected arm of one patient to capture the surface electromyography (EMG). The placement was confirmed with an inspection of EMG output recorded with Delsys, EMG works acquisition software. The EMG was sampled at 1926 Hz, amplified, and bandpass filtered at 20–450 Hz and was used to assess burst duration and the discharge pattern using EMG works analysis software. EMG signals were detrended with a 0.1 Hz high pass filter (3rd-order IIR filter) and rectified. Then a Hilbert transform was applied to the rectified EMG signals to obtain the amplitude envelope. The raw accelerometer signal was sampled at 148 Hz and filtered (0–50 Hz). Power spectral density analysis of a 30-second-long recording of acceleration signal was analyzed with the Welch method using 5-second epochs with no overlap (Figure 2). Spectral peak frequency, amplitude, and half-power bandwidth were computed offline. The peak spectral power for the tremor was calculated by squaring and summating the peaks of power in x, y, and z-axes and calculating the square root of the summated power. The half-peak bandwidth was the width of the spectral peak at one-half the peak amplitude in the power spectrum (a wider bandwidth of frequency peak indicating a more irregular tremor). We used the python software to compute the time-frequency spectra (spectrograms) of 25-second-long acceleration and EMG envelope signal (Figure 3).

**Figure 4:**
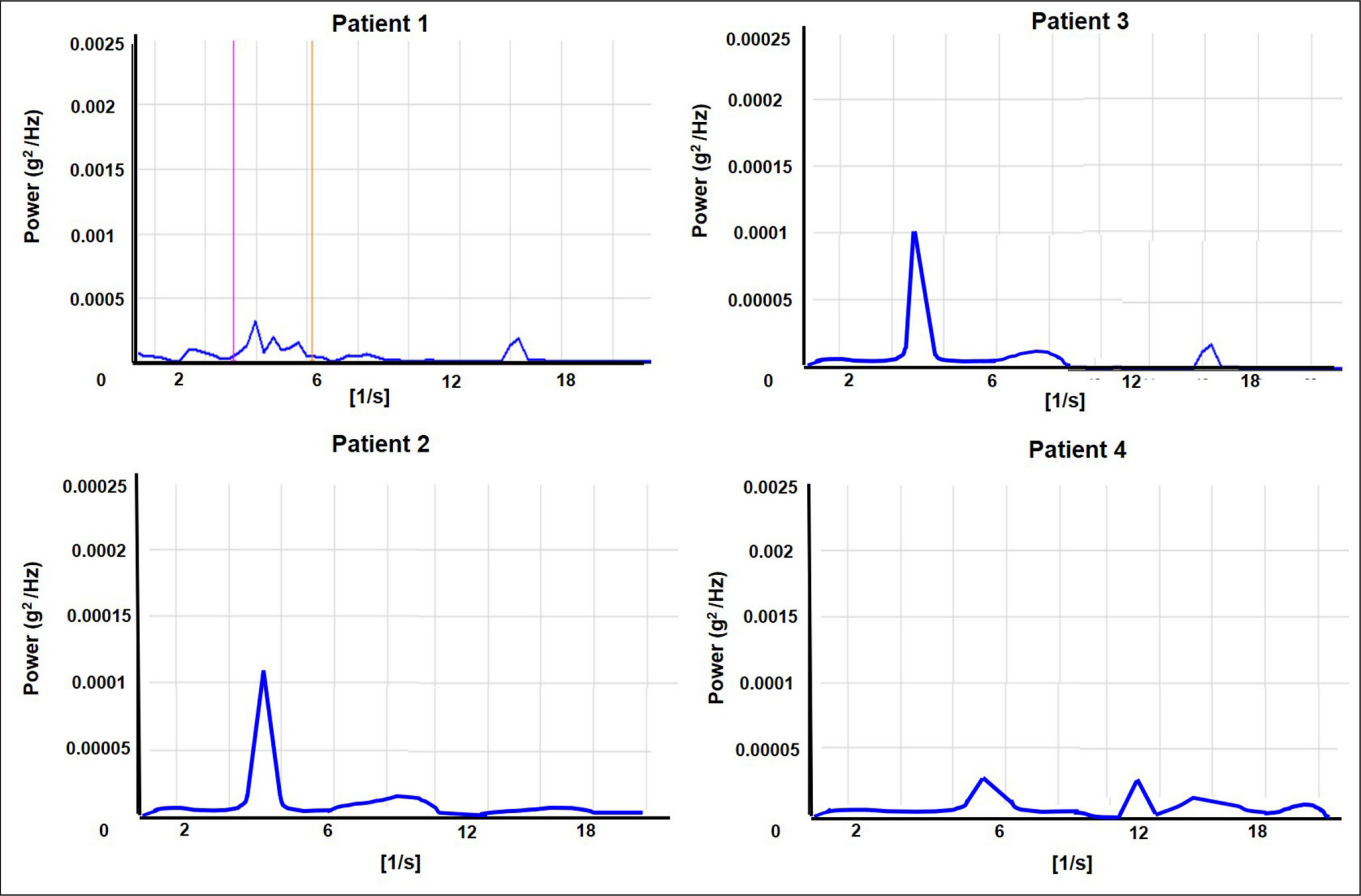
Power spectrum analysis of accelerometer signal recordings performed after limb cooling for each participant.

We performed a limb cooling test in which ice packs placed inside a thin pillowcase were wrapped around the forearm and hand. (Figures 1B and C) The icepacks were placed for 10 minutes to cool the skin surface to at least 20 degrees F below the baseline temperature, confirmed with a digital thermometer. The icepacks were then removed and the patients were asked to extend their arms and maintain a steady posture. We measured the tremor amplitude, frequency, and bandwidth with an accelerometer placed at the same location as before (Figure 4). We determined whether limb cooling significantly changed physiological parameters such as amplitude, frequency, and bandwidth using a nonparametric Wilcoxon signed-rank test.

## Case Presentations

### Patient 1

A 63 years old African American male diagnosed with end-stage renal disease (ESRD) secondary to Type II diabetes received a renal graft from a deceased donor four months prior to the presentation. Immediately after the surgery, he was initiated on a tacrolimus-based immunosuppressant regimen. Within a week of initiation of the medication, he noticed bilateral hand tremors manifesting when holding a coffee mug, smartphone, or television remote control. He endorsed difficulty eating and handling utensils with food dropping on many occasions. There was no tremor reported during resting position. Tremor did not involve other body parts. No comorbidities or alternative etiologies could explain the onset of tremor. At the time of assessment, he received tacrolimus at a total daily dose of 8 mg/day with a trough level of 5.4 ng/mL. Physical examination revealed a mild, distal, flexion-extension, rhythmic, postural and kinetic tremor affecting mainly the interphalangeal joints of the hand (supplemental video). Tremor was observed to increase when kinetic motor tasks like holding a cup, writing and drawing were performed. There was no rest or intentional component. (Table 1). The accelerometer analysis revealed a frequency of 5.5 Hz on the power spectrum analysis (Table 2), with harmonics recorded at higher frequencies due to the non-sinusoidal waveform of the recordings (Figure 2). Tremor was observed to have a narrow half-peak bandwidth (0.9 Hz). A power spectrogram revealed that there were no fluctuations in the tremor frequency (Figure 3). Tremor amplitude also reduced remarkably during limb cooling, and the peak frequency was observed to be broad and poorly defined (Figure 4).

**Supplemental Videos:** **Postural component recordings for tacrolimus induced tremor**. Videos were recorded during the postural elevation of arms task. Video segments for the individual participants reveal distal bilateral hand tremors. The tremor was mainly symmetric, involving the metacarpophalangeal and interphalangeal joints. Patient 3 had a slightly jerky tremor in the right hand during the arm elevation task however had a rhythmic tremor during kinetic tasks (not shown in the video).

**Table 2:** Physiological findings obtained from accelerometer-based recordings.


	PATIENT 1	PATIENT 2	PATIENT 3	PATIENT 4

**Postural tremor**				

frequency in Hz	5.5	5.5	5	6

amplitude in g^2^/Hz (mean ± SD)	0.006 ± 0.001	0.005 ± 0.001	0.04 ± 0.002	0.001 ± 0.001

bandwidth in Hz	0.9	1	1.5	1.3

**Effects of cooling**				

frequency in Hz	5.5	4.5	4	6

amplitude in g^2^/Hz (mean ± SD)	0.0001 ± 0.0001	0.0001 ± 0.0002	0.0002 ± 0.0002	0.00001 ± 0.00002

bandwidth in Hz	2.1	1.5	1.8	1.6


Mean of fundamental and harmonic power with standard deviation (SD) is provided.

### Patient 2

A 57 years old white female diagnosed with ESRD secondary to Autosomal Dominant Polycystic Kidney Disease received a renal graft from a deceased donor. Immediately after the surgery, she was placed on tacrolimus therapy. Within three weeks of starting the medication, she began to have difficulties with eating, drinking and writing due to tremors. Her right hand was more affected compared to her left hand. Physical examination three months later revealed mild, distal, flexion-extension, fine, rhythmic, postural, and kinetic tremors affecting the interphalangeal joints (supplemental video). There was no rest or intentional component (Table 1). At the time of evaluation, she took a total daily dose of 5 mg/day of tacrolimus. The tacrolimus trough level was 11 ng/mL. The accelerometer-based power spectrum analysis demonstrated a 5.5 Hz frequency with 1.2 Hz as half peak bandwidth (Table 2). Similar to patient 1, the power spectral analysis revealed high-frequency harmonics. A power spectrogram revealed that there were no fluctuations in the tremor frequency (Figure 3). The tremor decreased with limb cooling. There was slight change in the frequency and the half-peak bandwidth in response to limb cooling. (Figure 4).

### Patient 3

A 73 years old white male with ESRD secondary to focal segmental glomerulosclerosis received a kidney transplant from a living donor in 2008. Since surgery, he reported taking tacrolimus at a steady dose. His hand tremor surfaced shortly after the surgery (within weeks), impacting activities of daily living such as eating, writing, and fine motor tasks like using a screwdriver. He was prescribed metoprolol 100 mg/day to control tremors and blood pressure. He found partial improvements with the medication. At the time of his assessment, he took extended-release tacrolimus at a 2 mg daily dose. He reported switching to extended-release preparation five years after the surgery; however, he did not observe a notable improvement in tremor severity with the change. The tacrolimus trough levels at the time of his visit were 5.1 ng/mL.

Physical examination revealed a moderately severe, coarse, rhythmic flexion-extension hand tremor affecting the distal interphalangeal, metacarpophalangeal, and wrist joints (supplemental video). There was no rest or intentional component. The average EMG burst duration was around 75 ms. (Figure 2) An accelerometer assessment (off medication) revealed tremors of approximately 5 Hz frequency with a half peak bandwidth of 1.5 Hz. High-frequency harmonic peaks, as observed in the power spectrum analysis of accelerometer signals for patient 1 and patient 2 were seen but less pronounced. Tremor amplitude returned to baseline once the surface temperature increased to room temperature. A power spectrogram revealed that there were no fluctuations in the tremor frequency (Figure 3). Limb cooling reduced tremor amplitude with no significant change in frequency and half peak bandwidth (Figure 4).

### Patient 4

A 72 years old white male diagnosed with ESRD secondary to glomerulonephritis underwent kidney transplant surgery from a living donor ten years ago. In the beginning, he received cyclosporine for about six months which reportedly did not lead to tremors. He was switched to tacrolimus for better graft survival. Upon receiving tacrolimus at a dose of 6mg/day, he developed bilateral hand tremors within two weeks. He complained of handshaking when holding or pouring coffee or eating with a fork and spoon. The tremor persisted even after lowering the dose to 2 mg in the morning and 1.5 mg in the evening. At the time of evaluation, tacrolimus trough levels were 3.3 ng/mL. He endorsed partial improvements with metoprolol therapy at 50mg/day. Physical examination revealed mild to moderate severity, flexion-extension, and slightly coarse hand tremor involving the distal interphalangeal joints (supplemental video). The tremor affected the right side more compared to the left side. The tremor was kinetic > postural. There was no rest or intentional component. The electrophysiological assessment revealed a 6 Hz peak (no high-frequency harmonics) with a half peak bandwidth of 1.6 Hz. Limb cooling reduced tremor amplitude, which returned to baseline as the skin surface attained room temperature. A power spectrogram revealed that there were no fluctuations in the tremor frequency (Figure 3).

There was no significant change in frequency or half-peak bandwidth in response to limb cooling (Figure 4).

Nonparametric (Wilcoxon signed rank) statistical comparisons of the amplitude, frequency, and bandwidth of tremors before and after limb cooling revealed reduced amplitude with limb cooling (p = 0.03) but there was no change in frequency or bandwidth (p > 0.05).

## Discussion

Diagnosis of drug-induced tremors is based on a temporal relationship between drug administration and the emergence of tremors [1]. Tremor can involve any body part; however, the arms and hands are most commonly affected [1]. A tremor affecting less usual body parts such as the jaw or the lower limbs may also be detected in some individuals. The tremor usually is symmetric and nonprogressive.

Our cohort included two patients with tremors assessed early in the course after the onset and two with tremors present for several years. None of the patients had tremors before surgery or tacrolimus drug therapy. Tremors involved distal hands and were postural and kinetic, with the kinetic component being more pronounced in three out of four patients. The tremor was mild for three patients and moderately severe for one patient. None of the participants had a visible or symptomatic tremor in other body parts. A previous study reported a resting component in almost 50% of patients, with 40% having tremors in both upper and lower limbs and 24% having tremors involving the head and facial muscles [15]. Our patients did not reveal a resting component or tremor involving other body parts. As reported in a previous study, our patients did not complain of additional neurological side effects such as ataxia, dysarthria, seizures, and insomnia [9].

Our study is one of the few studies that characterizes the physiology of tacrolimus-induced tremors. According to the consensus classification, a tremor can be considered to have low (<4 Hz), medium (4–7 Hz) and high (>7 Hz) frequencies [16]. Unlike previous studies that found a higher frequency peak of around 8 Hz [17,18], our participants consistently revealed a medium range frequency peak of about 5.5 Hz and the fundamental frequency of oscillation was stable over time. As shown in the figure, the waveforms were non-sinusoidal, which is in keeping with the nonlinear nature of oscillators causing tremors. Such oscillators are prone to producing non-sinusoidal waveforms (“harmonic distortion”) with features resembling a saw-tooth, triangular, or square-wave oscillation. Another finding was related to the harmonics of fundamental tremor frequency seen in some of our participants, also previously described in Parkinson’s disease tremors [16,19]. In our opinion, these harmonics may be reflect a shift from the fundamental frequency to a subharmonic oscillation or super-harmonic oscillation.

The exact mechanisms underlying tacrolimus-induced tremors are not known. Tacrolimus is known to induce ataxia co-occurring with tremor symptoms [15]. Tremor and ataxia are likely attributable to the abnormalities in the cerebellar circuitry and the altered sensitivity profile of the GABA receptors [9]. To date, no study has examined the effects of limb cooling on tacrolimus-induced tremors which are important as they provide pathophysiological insights and an opportunity to develop effective therapies. In our study, while the effect of limb cooling on frequency was not statistically significant, tremor amplitude was observed to reduce in three of four patients. Limb cooling has been studied previously in essential tremor and has been found to lead to tremor suppression [20]. In another study, a bedside ice test appeared to have a differential effect on suppressing essential tremor compared to Parkinson’s disease tremor [21]. Limb cooling has been proposed to lower the muscle spindle sensitivity, reduce peripheral nerve conduction velocity, and increase joint stiffness [22]. We speculate that an altered kinesthetic feedback from the peripheral tissues delivered to the central source of oscillation may have led to amplitude reduction. These hypotheses will need validation in future studies.

The first reports of tacrolimus induced tremor emerged three decades ago when 36% of pediatric and 22% of adult patients undergoing orthotopic liver transplantation reported tremor with tacrolimus therapy [23,24]. The adult patients complained of a more severe action tremor that interfered with day-to-day tasks such as eating and writing. A lower dose reduced the tremor, although several patients continued to have mild, non-bothersome tremor. [23] Cyclosporine is another calcineurin inhibitor widely used in post-transplant patients’ immunosuppressive regimens. A review of cyclosporine neurotoxicity reported postural and intention tremors in up to 40% of patients [7]. The risk of tremor with cyclosporine has been found to be lower than with exposure to tacrolimus [3].

The usual risk factors for drug-induced tremors are older age, male sex, polypharmacy, administration of high doses and reaching toxic levels [1]. Conversion of tacrolimus to the extended-release formulation may lower risk of tremors, but one of our patients developed tremor on extended-release tacrolimus. In one study, common reasons patients were switched to extended-release tacrolimus were wide fluctuations in tacrolimus levels (44%) and to minimize the adverse effects of tremors (32%). Among patients who were switched due to tremors, 88% reported significant improvement in symptoms [25]. Another study with open-label assessments also reported similar tremor improvements when patients switched from immediate release to sustained release tacrolimus [18]. However, another study found no difference in the incidence of graft survival and side effects including tremor between patients receiving immediate release and extended release tacrolimus preparations [26].

There are no definitive therapies for treating tacrolimus-induced tremors. In general, removal of the offending drug leads to mitigation of tremor; however, the use of tacrolimus for a patient receiving a kidney transplant can be life-saving. Thus, discontinuing the drug may not be a practical solution. Instead, developing potent symptomatic pharmacological or nonpharmacological therapies may be prudent. Our assessments, albeit in a small cohort, found that limb cooling consistently yielded benefits. These findings are important as limb cooling is noninvasive, easy to implement, and could be recommended to patients on immunosuppressants. Another option to consider for treating tacrolimus-induced tremors is using adaptive devices, for example, heavy writing devices often recommended to patients with essential tremor to help cope with writing [27]. Finally, regarding pharmacological options, two patients in our cohort who had long-duration tremors received metoprolol for dual control of tremors and elevated blood pressure which is similar to what is done in essential tremor [28]. These patients endorsed only partial improvements. Future larger cohorts that examine the role of limb cooling, weighted adaptive devices, and the use of beta-blockers and GABAergic drugs will further confirm the therapeutic potential of these individual interventions.
